# Structure-aware completion of plant 3D LiDAR point clouds via a multi-resolution GAN-inversion network

**DOI:** 10.3389/fpls.2025.1698843

**Published:** 2025-12-19

**Authors:** Zhiming Wei, Jianing Long, Zhihong Zhang, Xinyu Xue, Yitian Sun, Qinglong Li, Wu Liu, Jingxin Shen, Zhikai Zhang, Xiaoju Li, Zhengguo Ma

**Affiliations:** 1 Nanjing Institute of Agricultural Mechanization, Ministry of Agriculture and Rural Affairs, Nanjing, China; 2 Shandong Academy of Agricultural Machinery Sciences, Jinan, China; 3 Huang Huai Hai Key Laboratory of Modern Agricultural Equipment, Ministry of Agriculture and Rural Affairs, Jinan, China; 4 College of Information and Telecommunications, China Agricultural University, Beijing, China; 5 Faculty of Intelligence Technology, Shanghai Institute of Technology, Shanghai, China; 6 Bureau of Agriculture and Rural Affairs of Yanggu County, Liaocheng, China; 7 College of Mechanical and Electrical Engineering, Qingdao Agricultural University, Qingdao, China

**Keywords:** plant canopy architecture, 3D plant modeling, point cloud completion, deep learning, multi-resolution, GAN inversion

## Abstract

**Introduction:**

Three-dimensional (3D) point clouds acquired by LiDAR are fundamental for applications such as autonomous navigation, mobile robotics, infrastructure inspection, and cultural-heritage documentation. However, environmental disturbances and sensor limitations often yield incomplete or noisy point clouds, degrading downstream performance. This study addresses robust, high-fidelity point cloud completion under such practical conditions.

**Methods:**

We propose an unsupervised deep learning framework, Multi-Resolution Completion Net (MRC-Net), which builds on ShapeInversion by integrating a Generative Adversarial Network (GAN) inversion strategy with multi-resolution principles. The architecture comprises an encoder for feature extraction, a generator for completion, and a discriminator to assess geometric integrity and detail. Two key designs enable strong performance without supervision: (i) a multi-resolution degradation mechanism that guides reconstruction across coarse-to-fine scales, and (ii) a multi-scale discriminator that captures both global structure and local details.

**Results:**

Extensive experiments on multiple datasets demonstrate that MRC-Net achieves accuracy comparable to leading supervised approaches. On virtual datasets (e.g., CRN), MRC-Net attains an average Chamfer Distance (CD) of 8.0 and an F1 score of 91.3. On a custom dataset targeting agricultural scenarios, the model preserves object integrity across varying complexity: for regular cartons, it achieves CD 3.3 and F1 97.3; for structurally complex simulated plants, it maintains overall shape while delivering average CD 8.6 and F1 88.1.

**Discussion:**

These results indicate that MRC-Net advances unsupervised point cloud completion by balancing global shape consistency with fine-grained detail. The method provides a reliable data foundation for downstream tasks—including autonomous navigation, high-precision 3D modeling, and agricultural robotics—thereby contributing to improved data quality in precision-agriculture and related domains.

## 1 Introduction

The important role of 3D point cloud data in representing object shapes and environmental structures is well-recognized across a myriad of fields, including agricultural computer vision, robotics, and autonomous driving (Li et al., 2022; Tang et al., 2024; Huang et al., 2025; Li et al., 2025a; Qiao et al., 2025). However, real-world scanning processes, particularly those involving Light Detection and Ranging (LiDAR) technology, often yield incomplete or partially observed point clouds due to factors like occlusions, limited fields of view, and sensor noise (Yan et al., 2022; Khorsandi et al., 2023; Wang et al., 2025a, Wang et al., 2025b). This data incompleteness presents a significant bottleneck, as it can severely impair the effectiveness of downstream tasks such as object detection, segmentation, and recognition.

These challenges are particularly consequential within the realm of precision agriculture. Technologies such as variable-rate application depend on detailed 3D field maps to enable the precise delivery of inputs like pesticides (Zhu et al., 2017; Fessler et al., 2021; Wang et al., 2023), herbicides (Andújar et al., 2013), or fertilizers (Zhao et al., 2021; Cruz Ulloa et al., 2022). Point cloud data is invaluable for creating prescription maps by capturing critical metrics like plant height, density, and volume (Abbas et al., 2020; Gu et al., 2020; Zeng et al., 2020; Zhao et al., 2020; Wang et al., 2022). Yet, when the underlying point clouds are incomplete, the accuracy of the prescription maps is compromised, thereby limiting the precision of agricultural inputs (Zhang et al., 2023). Therefore, the field of point cloud completion—which focuses on estimating and integrating missing data—is of paramount importance.

Point cloud completion technologies predominantly fall under two methodologies, supervised and unsupervised learning. Given the inherent limitations of real-world scanning data, the potential for geometric structure loss, and the challenges that supervised learning faces in these circumstances, the exploration and advancement of unsupervised solutions become critical. This need underscores the importance of continuous research in this field. To elaborate, supervised and unsupervised learning, the two primary methodologies in point cloud completion tasks, each present unique advantages and limitations when applied to the process of completion. Supervised approaches (Dai et al., 2017b; Dai et al., 2018; Mo et al., 2019; Zhang et al., 2020), typically necessitate paired data sets of incomplete and complete point clouds for training. Eigen et al. (2014) pioneered the recovery of voxel-based depth maps using a single image. Choy et al. (2016) proposed the 3D-R2N2 Encoder3D LSTM-Decoder to model the mapping from 2D images to 3D voxels. Subsequently, the 3D-Encoder-Predictor Networks (3D-EPN) introduced by Dai, et al. (2017b) was used to predict the missing parts of point clouds. The PointNet presented by Qi et al. (2017a) addressed the issue of point cloud disorder, while Yang et al. (2017) enabled 3D reconstruction from a single image and direct output of point cloud coordinates. Achlioptas et al. (2018) proposed a L-GAN model for generating depth in point clouds, and PCN developed by Yuan et al. (2018) was pioneered to facilitate shape completion based on deep learning. The PF-Net presented by Huang et al. (2020) introduced the Generative Adversarial Network (GAN) to generate complete point clouds, though it was limited to filling only spherical missing parts. Techniques that progress from coarse to fine detail (Huang et al., 2020; Liu et al., 2020; Wang et al., 2020; Xie et al., 2020) have shown significant results in 3D virtual datasets. Despite their theoretical utility, these methodologies present practical challenges in real-world 3D completion applications, primarily due to their need for paired incomplete-complete datasets for training. Furthermore, their generalization capacity is often limited, constraining the applicability of these methods to diverse or unanticipated scenarios. These limitations necessitate further advancements in the field to increase robustness and versatility of point cloud completion techniques.

Considering the intrinsic constraints of LiDAR in obtaining exhaustive real-world scanning data—making it less compatible with conventional supervised learning methods—an unsupervised learning-based approach is proposed for 3D point cloud completion. This approach aims to mitigate the dependency on complete data sets and enhance the model’s capacity to generalize to diverse and complex real-world applications. For instance, Chen et al. (2019) proposed pcl2pcl that employs adversarial training to complete unpaired point clouds. Further, RL-GAN Net proposed by Sarmad et al. (2019) integrates Reinforcement Learning (RL) with Generative Adversarial Networks (GAN) to enhance robustness and accelerate prediction. Additionally, ShapeInversion proposed by Zhang et al. (2021) was the first to incorporate GAN inversion into the process of 3D shape completion.

More recently, unsupervised and generative‐prior frameworks have advanced label-free point cloud completion. Lee et al. (2024) cast unpaired completion as an unbalanced optimal transport problem (UOT-UPC), improving robustness under class imbalance and without paired supervision. Complementarily, diffusion-based priors enable strong generalization: Huang et al. (2024) complete partial shapes at test time using 2D diffusion guidance with no additional training; Wei et al. (2025) leverage multi-view diffusion priors with fusion and consolidation to recover both global symmetry and thin structures; and Zhang and Liu (2024) introduce an occupancy-diffusion scheme with a coarse-to-fine design. To further enhance cross-domain robustness, Li et al. (2025b) propose DAPoinTr, a domain-adaptive Point Transformer that narrows distribution gaps and stabilizes predictions across datasets. Notwithstanding, existing unsupervised point cloud completion methods often primarily focus on the overall shape completion, while detailed structural aspects tend to be overlooked. This discrepancy poses a significant challenge as it may lead to the loss of essential, intricate details in the completion process.

To address the aforementioned challenges, this study proposes the Multi-Resolution Completion Network (MRC-Net), an unsupervised framework for 3D point cloud completion that preserves global structure while recovering fine-grained details by coupling a multi-resolution degradation mechanism with multi-scale discriminators. The approach introduces a detail-aware completion architecture that jointly models global geometry and local structures to complete imperfect, LiDAR-like inputs without paired supervision; employs a principled training strategy that synthesizes realistic partial observations (e.g., occlusion, sparsity, and noise) and applies multi-scale adversarial guidance to enhance fidelity and robustness across resolutions; and is validated through comprehensive experiments on synthetic benchmarks (e.g., CRN) and real-world agricultural datasets, including comparisons to recent baselines and ablation studies, demonstrating strong generalization under diverse levels of incompleteness and noise.

## 2 Materials and methods

### 2.1 Construction of point cloud completion network model

#### 2.1.1 The application of MRC-net for point cloud completion

**Figure 1:**
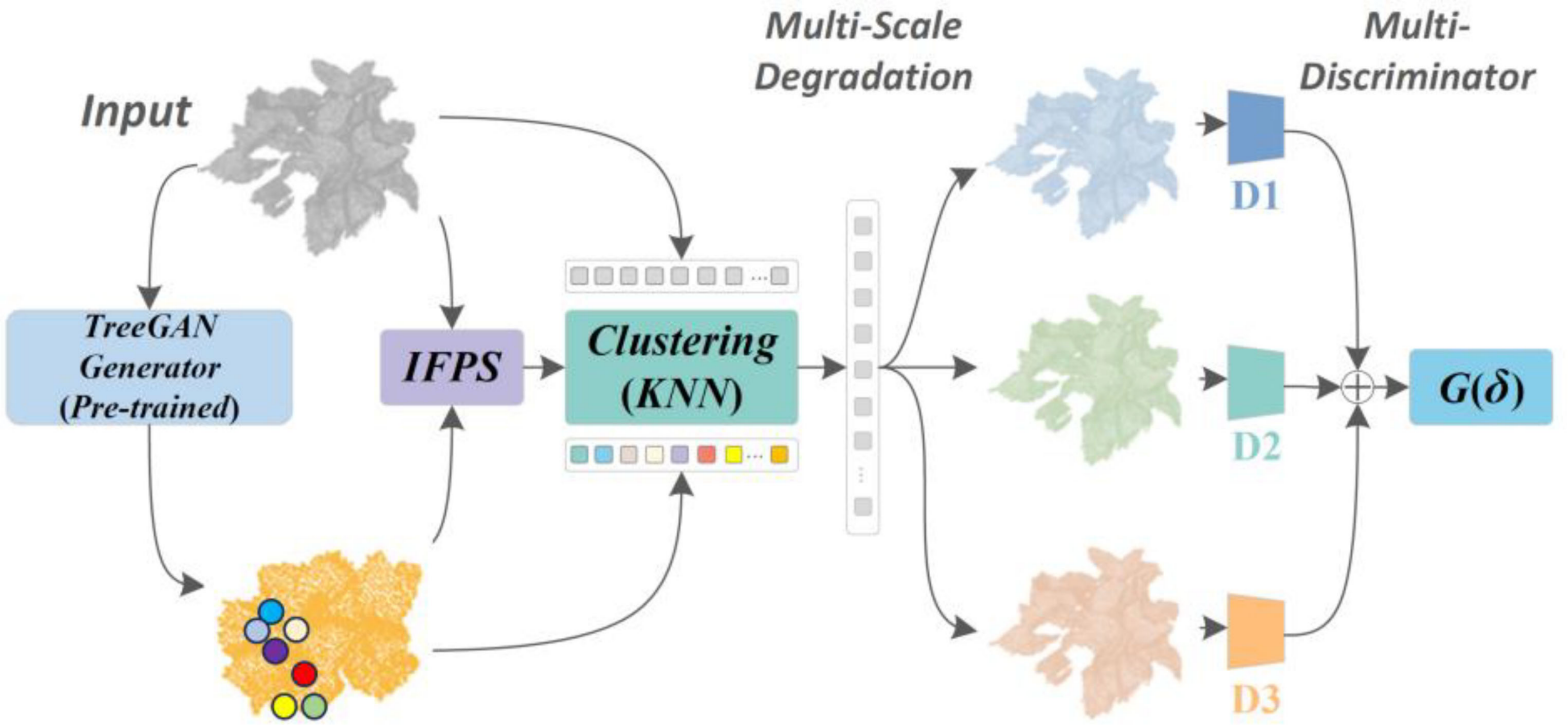
Unsupervised MRC-net application for 3D point cloud completion.

In this investigation, a methodology was developed which broadens the foundational work of ShapeInversion, addressing the intricate challenges presented by 3D point cloud completion. The procedural flow, as illustrated in **Figure 1**, is detailed as follows. The procedure of feature extraction started by extracting features from both complete and incomplete point clouds. The generator was initially trained to produce coarse point cloud data, which was subsequently degraded to resemble the input point cloud at varying resolutions. Multiple discriminators, guided by a specific loss function, further instructed the generator to create more realistic point clouds. In pre-training and initial generation, the model was pre-trained on a set of complete point cloud models to facilitate the extraction of latent vectors from the latent space. These vectors, encompassing vital features, enabled the generator (*G*) to produce an approximate complete point cloud. In multi-resolution down sampling, the Iterative Farthest Point Sampling (IFPS) method is employed to down sample the roughly generated complete point cloud into three point clouds with different resolutions: $X_{a},\;\;X_{a}^{'},\;\;and\;X_{a}^{''}$ (consisting of 2048, 1024, and 512 points respectively). In degradation using multi-resolution mechanism, a mechanism known as Mk-Mask, perceptible as a clustering task using the K-Nearest Neighbor (KNN) method, degraded these point clouds at various resolutions into shapes akin to the incomplete input *X*_in_, resulting in $X_{b},\;\;X_{b}^{'},\;\;and\;X_{b}^{''}$, respectively. In progressive optimization and evaluation, the generator was progressively fine-tuned through a multi-stage reconstruction loss and multi-scale feature matching loss. This optimization process involves the MRC-Net matching a set of latent codes in the pre-trained GAN’s latent space, from which a complete point cloud structure was reconstructed. The completed point cloud was then subdivided and degraded into incomplete structures via IFPS and KNN, concluding with the integration of multi-discriminators to enhance the generator. The performance of the generation network was evaluated in the final step. This methodological framework served as a robust analytical approach, extending existing point cloud completion models, and set the stage for the development and refinement of techniques tailored to the intricate nature of 3D point cloud completion.

**Figure 2:**
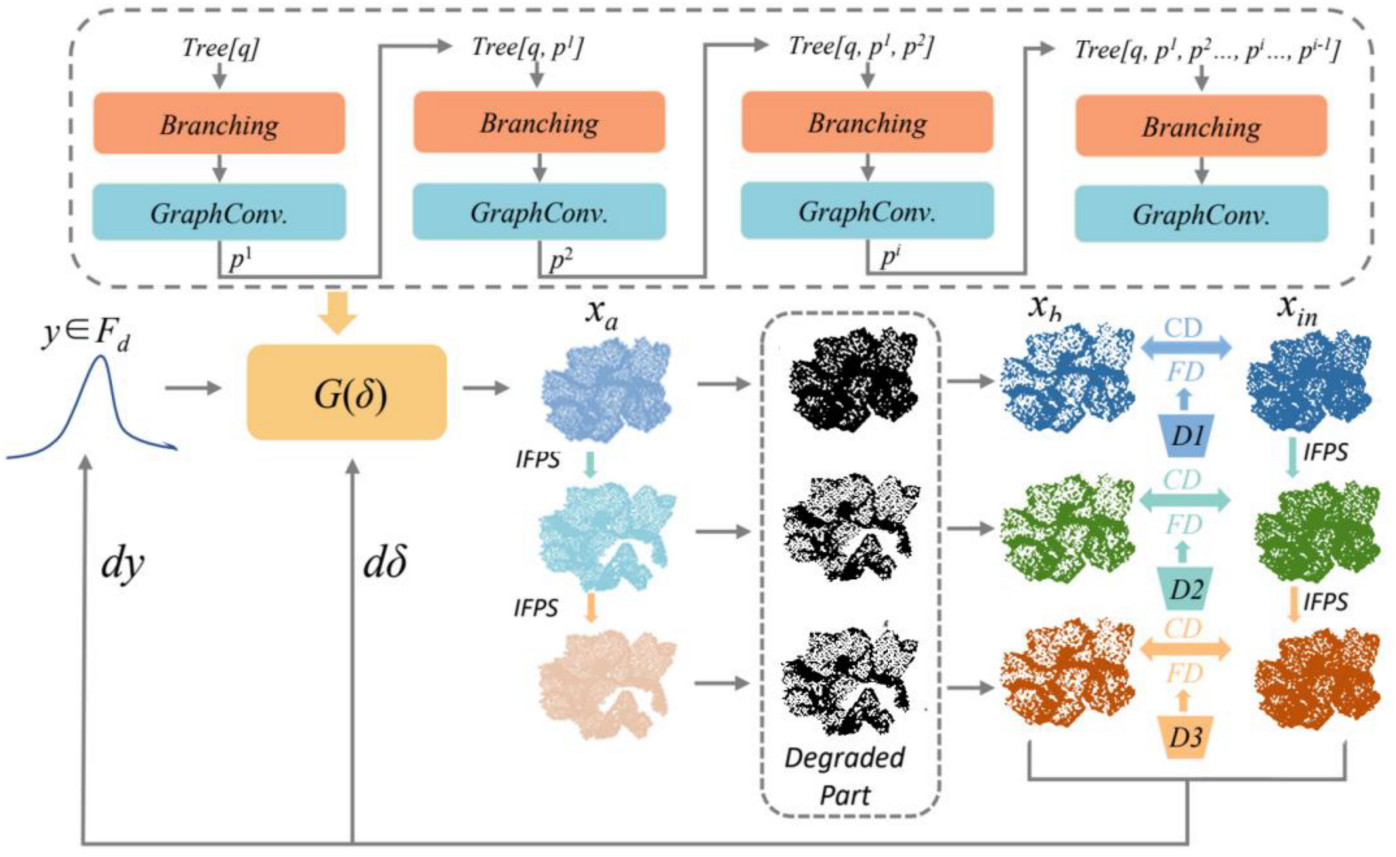
Illustration of the MRC-net’s strategy: seeking latent codes ‘y’ within the hidden space of a pre-trained GAN and incrementally updating the parameters ‘*δ*’ of the generator ‘*G*’ to enhance the quality of generation.

In addressing incomplete point cloud data acquisition by LiDAR sensors, the MRC-Net was introduced in this study. MRC-Net, illustrated in **Figure 2**, predicts the comprehensive structure of point clouds from incomplete depictions, adapting the GAN inversion concept for 3D structure completion. The architecture primarily incorporates a multi-scale degradation network Mk-Mask and an optimization network. Initially, the generator, collaborating with IFPS, fabricates multi-resolution point clouds. These are then degraded to mirror the incomplete input *X*_in_ via the multi-scale degradation mechanism. The process culminates with the optimization of the generator using a progressive, multi-stage reconstruction loss in combination with a multi-scale feature matching loss, followed by an evaluation of the resultant output.

#### 2.1.2 Adaptive GAN inversion for enhanced 3D point cloud completion and multi-resolution reconstruction

In 3D point cloud completion tasks, GAN networks typically employ a generator to capture latent vectors, which are further used to accomplish the generation task. Traditional GAN networks, despite having dynamically changing input parameters, usually employ a fixed generator architecture. A pre-trained GAN was used on a set of complete point clouds from a particular class to obtain a collection of effective prior knowledge containing rich 3D geometric structure and semantic information. The generator exploits the latent vectors from this prior knowledge to generate complete point cloud models. Furthermore, the generator is allowed to utilize a combination of latent vectors *y* and generator parameters *δ* for real-time fine-tuning and continuous optimization of the generator. Applying GAN inversion to point cloud completion tasks enables the generator G to identify the optimal latent vectors for reconstructing complete point cloud models.

During the pre-training phase, a generator parameterized by *δ* is trained on complete point cloud models. In this study, the pre-trained generator is used to generate shape $X_{a}\in F_{m}\times 3$ from latent vectors $y\in F^{d}$. The incomplete input point cloud model Xin can be reconstructed into a complete point cloud model closely resembling the ground truth through G, as shown in (Equation 1):

(1) $$\begin{array}{c} y^{*}=\underset{y\in F^{d}}{\arg\min}E\left(G\left(y;\;\delta \right),\;X_{in}\right),\;X_{a}^{*}=G\left(y^{*};\;\delta \right)\; \end{array}$$

An approach inspired by GAN inversion for image generation in 2D images (Pan et al., 2021) has been adopted to achieve improved completion results with the generator G. For each point cloud, the generator is adapted by updating the generator parameters δ, while the latent vectors y was concurrently optimized for the progressive fine-tuning of the generator. This was represented in (Equation 2).

(2) $$\begin{array}{c} \delta ,\;y^{*}=\underset{y,\;\delta }{\arg\min}E\left(G\left(y;\;\delta \right),\;X_{in}\right)\; \end{array}$$

The GAN inversion initialization began with an extensive random sampling of latent vectors. The latent vector associated with the smallest ϵ value was then chosen as the initial parameter for fine-tuning the generator in relation to y. In real-time, the parameters y and δ are updated using gradient descent as illustrated in (Equation 3). Subsequently, the generator integrated with the latent vectors to construct a complete Xa from the incomplete input Xin. Leveraging an approach inspired by FPN (Lin et al., 2017), the complete point cloud structure Xa was then merged with the multi-resolution degradation mechanism Mk-Mask. This process ultimately leads to the transformation of the complete 3D model into point cloud models of diverse resolutions, analogous to the input.

(3) $$\begin{array}{c} y^{*}=\underset{y\in R^{d}}{\arg\min}E\left(N(G(y;\;\delta )),\;X_{in}\right)\; \end{array}$$

#### 2.1.3 Efficient feature extraction from point cloud data using iterative farthest point sampling

**Figure 3:**
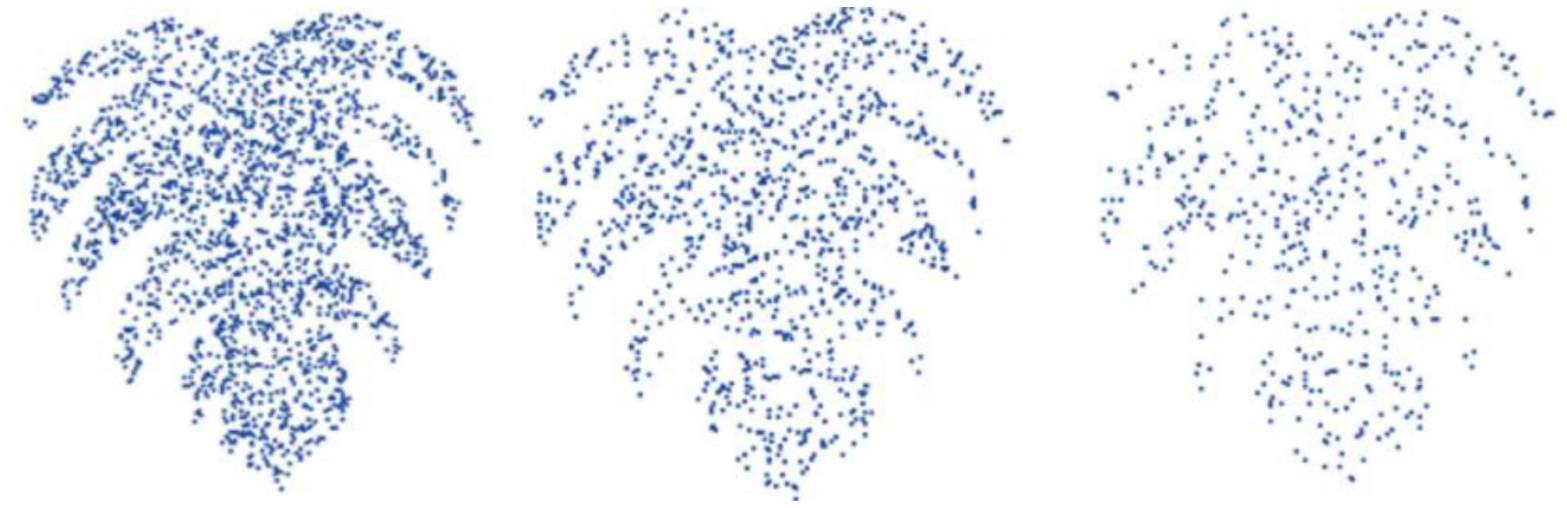
Demonstration of point clouds at varied resolutions.

This study extracted a limited number of key feature points from large quantities of point cloud data to efficiently represent the model’s structural information, thus enhancing the computational efficiency of deep learning networks. The IFPS method, as utilized in PointNet++ (Qi et al., 2017), was employed to extract the principal feature points from the point cloud data. This method, despite its increased computational complexity compared to uniform and random sampling, provided a more uniform distribution of sampled points across the entirety of the model’s point set. In **Figure 3**, point clouds at various resolutions were depicted. It became evident that even as the point quantity decreases in low-resolution point clouds, the fundamental structural features of the 3D point cloud were still adequately captured. The application of the IFPS method ensures computational efficiency while preserving essential geometric and semantic information. Notably, even in reduced-resolution representations, the structural integrity of the 3D point cloud was maintained. Through the use of IFPS, an equilibrium was struck between computational demands and the preservation of crucial geometric and semantic aspects.

#### 2.1.4 Multi-resolution degradation mechanism component in point cloud completion

The generator used latent vectors to create a complete point cloud structure, denoted as *X*_a_ = G(*δ*). Through the application of IFPS, initial point cloud shapes of varying resolutions were produced. The multi-resolution degradation mechanism, a variant of the recent degradation transformation module k-Mask (Zhang et al., 2021), referred to as Mk-Mask in this study, served as the initial comparator for the multi-resolution incomplete point cloud model. Given the irregular and unstructured nature of point clouds in space, variations in spatial position information could occur within the same point cloud. Consequently, a direct degradation of the complete point cloud into the desired shape was not feasible via the degradation function alone. The Mk-Mask mechanism addressed this challenge by adopting ideas from the KNN algorithm, used for representing spatial-local correlations in 2D classification tasks, and from the Point CNN (Dai et al., 2017b) feature extractor, used for 3D point cloud tasks. The Mk-Mask was not confined to the degradation of generator-produced point clouds at a single resolution but could simultaneously degrade point clouds across multiple resolutions. Specifically, it performed a clustering task to group generated points into different resolutions, preserving the structure and information of the point cloud. The KNN algorithm aided in capturing the local structure of the point cloud by considering each point’s nearest neighbors. In contrast, Point CNN extracted hierarchical features from point clouds. By integrating these concepts, the Mk-Mask degraded the generated point cloud structures at multiple resolutions to more closely approximate the incomplete input point cloud. This mechanism was pivotal in ensuring alignment between the generated point clouds and the real-world structures and semantics represented by the incomplete input data, contributing to the generation of more accurate and realistic point cloud completions.

The IFPS method was employed to sample the generator-produced point cloud into various resolutions, denoted as *F*_i_, with sizes of 2048, 1024, and 512 for *i* = 1, 2, 3, respectively. This strategy enabled the multi-resolution degradation module, Mk-Mask, to concentrate on essential feature points. Subsequently, Mk-Mask degraded the multi-resolution complete point clouds into forms akin to the incomplete input, *X*_in_. Specifically, the degradation through *F*_3_ to *MF*_3_ primarily ensured the effect on the backbone structures. The transition from *F*_2_ to *MF*_2_ predominantly safeguards the secondary overall structures, while *MF*_1_ chiefly focused on details. By adopting a multi-resolution degradation approach, it was possible to extract more features pertinent to both high-resolution structures and semantics and low-resolution structures and semantics compared to single-resolution degradation when calculating feature distance losses. This procedure facilitated the generator to produce the most effective point cloud completion by using the optimal solution. Mk-Mask’s utilization of multi-resolution degradation was crucial to capturing different levels of detail in the point cloud. The mechanism allowed for the preservation of a wide array of features, formed primary structural components to finer details.

Furthermore, a methodology inspired by K-nearest neighbors (KNN) was implemented in this investigation to enhance the precision and robustness of the degradation mechanism when dealing with incomplete point clouds, represented as *X*_in_. This approach uses Euclidean distance as the metric for proximity assessment. Each point within *X*_in_, designated as *b*_i_, is assigned a neighborhood defined by a parameter K and compared against the complete point cloud, *X*_a_, produced by the generator. The collection of points within *X*_a_ located within the K-defined neighborhood relative to *X*_in_ are represented as $M_{k}^{x_{a}}\left(b_{i}\right)$. By executing this procedure for every point within the point cloud, the complete point cloud generated by the generator, *X*_b_, is gradually degraded into a configuration that closely mirrors the incomplete input *X*_in_. Therefore, as shown in (Equations 3, 4), *X*_b_ is composed of the aggregate of points encapsulated within these K-defined vicinities.

(4) $$\begin{array}{c} X_{b}=\overset{n}{\underset{i=1}{\cup }}\left\{U_{j}\in M_{k}^{X_{a}}\left(b_{i}\right)|b_{i}\in X_{in}\right\}\; \end{array}$$

#### 2.1.5 Integrated loss function components of the MRC-net

The loss function of the MRC-Net integrated two distinct elements, the multi-stage reconstruction loss and the multi-scale feature distance loss. The former, the reconstruction loss, functioned to measure discrepancies between the generated point cloud and the known ground truth. Meanwhile, the latter, the feature distance loss, employed a discriminator, which was trained in tandem with the generator, for feature matching within the feature space, with the objective of maintaining semantic and structural consistency. The calculation of the feature distance loss was facilitated through the utilization of the *L*_1_ distance metric. Together, these two interlinked elements of the MRC-Net’s loss function guide the model effectively towards accurate point cloud completion.

##### 2.1.5.1 Multi-stage reconstruction loss and the use of chamfer distance in point cloud completion

In the sphere of point cloud completion, two commonly utilized metrics for assessing the effectiveness of point cloud structure reconstruction were CD and Earth Mover’s Distance (EMD). Despite EMD’s heightened sensitivity towards capturing finer detail in point cloud generation, it exhibited limitations in our context since the training process entails degrading the complete shape, which was generated, to a form analogous to the input incomplete point cloud for comparison purposes. It was important to note that the number of points in the degraded shape may diverge from that in the input incomplete point cloud. Conversely, CD, being differentiable, supported more efficient computation. Hence, CD is chosen as the metric for reconstruction loss, as illustrated in (Equation 5).

(5) $${Ɛ}_{CD}\left(X_{b},X_{in}\right)=\frac{1}{\left|X_{b}\right|}\sum_{b\in X_{b}}\underset{c\in X_{in}}{\min}∥b-c{∥}_{2}^{2}\;+\frac{1}{\left|X_{in}\right|}\sum_{c\in X_{in}}\underset{b\in X_{b}}{\min}∥c-b{∥}_{2}^{2}$$

The CD was used to determine the mean shortest point distance between the degraded point cloud, *X*_a_, and the incomplete input point cloud, *X*_in_. Two versions, CD-P and CD-T (Dai et al., 2017a), support the calculation of the average closest point distance between the generated and real point clouds. CD-P was defined as $CD-P=\left(\sqrt{L_{X,Y}}+\sqrt{L_{Y,X}}\right)/2$, while CD-T is expressed as $CD-T=E_{X,Y}+E_{Y,X}$. In all training phase experiments conducted within this study, CD-T was utilized, which involves the computation of the L2 distance.

Utilizing the IFPS method, the incomplete input *X*_in_ is partitioned into two novel sets: $X_{in}^{'}$ and $X_{in}^{''}$, serving as the incomplete input feature points of size N3. Further, after GAN inversion, the complete point cloud was sampled via IFPS into three point clouds at disparate resolutions, each of size N3, analogous to the incomplete input, labeled as $X_{b}$, $X_{b}^{'}$, and $X_{b}^{''}$.

As a result, the multi-stage reconstruction loss is assembled from three components: ${Ɛ}_{CD1}$, ${Ɛ}_{CD2}$, and ${Ɛ}_{CD3}$. These are weighed by the hyperparameters *α* and *β*, as per (Equations 6). ${Ɛ}_{CD1}$, the first term, determines the squared distance between the refined points of the degraded point cloud *X*_b_ and the actual incomplete input *Xin*. The remaining terms, ${Ɛ}_{CD2}$ and ${Ɛ}_{CD3}$, calculate the squared distances between the primary structure points $X_{b}^{'}$ and secondary structure points $X_{b}^{''}$, and their corresponding real incomplete inputs $X_{in}^{'}$ and $X_{in}^{''}$. Thus, the multi-stage reconstruction loss is formulated as per (Equation 6).

(6) $$\begin{array}{c} T_{C}={Ɛ}_{CD1}\left(X_{b},X_{in}\right)+\alpha {Ɛ}_{CD2}\left(X_{b}^{'},X_{in}^{'}\right)+\beta {Ɛ}_{CD3}\left(X_{b}^{''},X_{in}^{''}\right) \end{array}$$

##### 2.1.5.2 Adopting feature matching loss and multi-scale discriminators for 3D point cloud tasks

Inspired by AniGAN’s (Li et al., 2021) use of feature matching loss for 2D image tasks to yield more natural data across various dimensions, and the proposed use of multiple GAN discriminators for a single image in 2D image tasks (Durugkar et al., 2016), this study adopted an analogous approach for 3D point cloud tasks. While the reconstruction loss function primarily emphasizes the overall structure, it failed short in finely detailing the completion process. To ameliorate this, L1 distance in the discriminator feature space was employed as a distance metric, aiming to optimize the generation of network details via discriminator’s distance metrics. Throughout the experimentation process, three discriminators, D1, D2, and D3, with identical network structures, were deployed. These discriminators operate on different point cloud resolutions, as determined by the multi-scale degradation mechanism. The choice of discriminators learned the method employed in 2D image tasks (Pan et al., 2021), where discriminators pretrained alongside the generator for the same task were used. As these discriminators exhibited high compatibility with the generator, they could finely tune the generator’s output distribution, thereby enhancing the quality of the results. This setup was reflected in (Equation 7).

(7) $$\begin{array}{c} T_{D}=\|D_{1}\left(X_{b}\right)-D_{1}{\left(X_{in})\right\|}_{1}+\|D_{2}\left(X_{b}^{'}\right)-D_{2}{\left(X_{in}^{'})\right\|}_{1}+\|D_{3}\left(X_{b}^{''}\right)-D_{3}{\left(X_{in}^{''})\right\|}_{1} \end{array}$$

The overall loss function is as shown in (Equation 8):

(8) $$\begin{array}{c} T=h_{C}T_{C}+h_{D}T_{D} \end{array}$$

The total loss function, as applied in this study, had an important role in both the generation and reconstruction of the complete shape. This function consisted of two major components, the reconstruction loss, weighted by *h*_C_, and the feature matching loss, weighted by *h*_D_. A crucial aspect of these weight parameters was that their sum was constrained to equal one, ensuring a balanced influence of both components on the overall loss. This restriction ensured that the overall loss function accurately reflects the interplay between the reconstruction and feature matching aspects, thus effectively guiding the process of point cloud completion.

#### 2.1.6 Comparison to baseline and design rationale

This subsection contrasts the proposed MRC-Net with the canonical baseline adopted in this study and clarifies the rationale behind each design choice. The baseline is taken to be a single-resolution encoder–decoder trained with reconstruction losses on synthetically partialed inputs. By comparison, MRC-Net is designed to address the principal failure modes encountered in real LiDAR scans—occlusion, sparsity/limited field of view, and measurement noise—through coordinated changes to input modeling, network architecture, and learning objectives. At the level of input modeling, MRC-Net employs a multi-resolution degradation mechanism that synthesizes LiDAR-like partial observations across several scales by stochastically composing occlusion masks, sparsity schedules, and noise injections. This broadens the training distribution to better match the statistics of real-world partial scans and thereby improves robustness without requiring paired supervision. In contrast, the baseline’s single-resolution partialing under-represents difficult cases such as large self-occlusions or highly anisotropic sparsity, which often leads to brittle behavior at test time. Architecturally, MRC-Net adopts a detail-aware global–local design that aggregates multi-resolution features to preserve coarse, topology-stabilizing structure while recovering high-frequency details. The coupling of global geometry with local structural cues helps retain symmetry and volumetric plausibility and, at the same time, reconstructs thin and highly curved parts. The baseline’s single-scale pathway tends to either over-smooth these fine structures or hallucinate disconnected fragments when incompleteness is severe. In terms of the learning objective, MRC-Net augments standard reconstruction losses with multi-scale adversarial guidance and geometric-consistency regularizers that align normal and regulate local curvature across resolutions. These additions encourage shape plausibility and suppress both over-smoothing and implausible hallucination under heavy missingness and noise. By comparison, reconstruction-only training in the baseline frequently trades detail fidelity for numerical stability, yielding overly conservative completions.

### 2.2 Dataset selection and preprocessing of point cloud completion based on MRC-net

#### 2.2.1 Dataset composition, preprocessing, and configuration for model evaluation

This study employed both synthetic and real-world scan datasets to rigorously evaluate the performance of the model across various contexts. For synthetic data, the baseline dataset, CRN (Wang et al., 2020), was used, selecting and annotating models from eight categories: chair, car, cabinet, couch, lamp, plane, table and watercraft. These models were organized in a common directory. The dataset was split as follows: 60% for training, 20% for validation, and 20% for testing. The input point cloud data undergo common preprocessing procedures, such as normalization of coordinate scales to the range [-1, 1], and recentering them around the origin. Both complete and incomplete point clouds within the synthetic dataset comprise 2048 points, with the incomplete point clouds forming subsets of their complete counterparts. Real-world scan datasets mirror the ones employed in ShapeInversion (Zhang et al., 2021) and pcl2pcl (Chen et al., 2019), which includes (1) KITTI (cars) (Geiger et al., 2012), (2) ScanNet (chairs and tables) (Dai et al., 2017a), and (3) MatterPort3D (chairs and tables) (Chang et al., 2017).

#### 2.2.2 Data acquisition, preprocessing, and augmentation for point cloud samples

**Figure 4:**
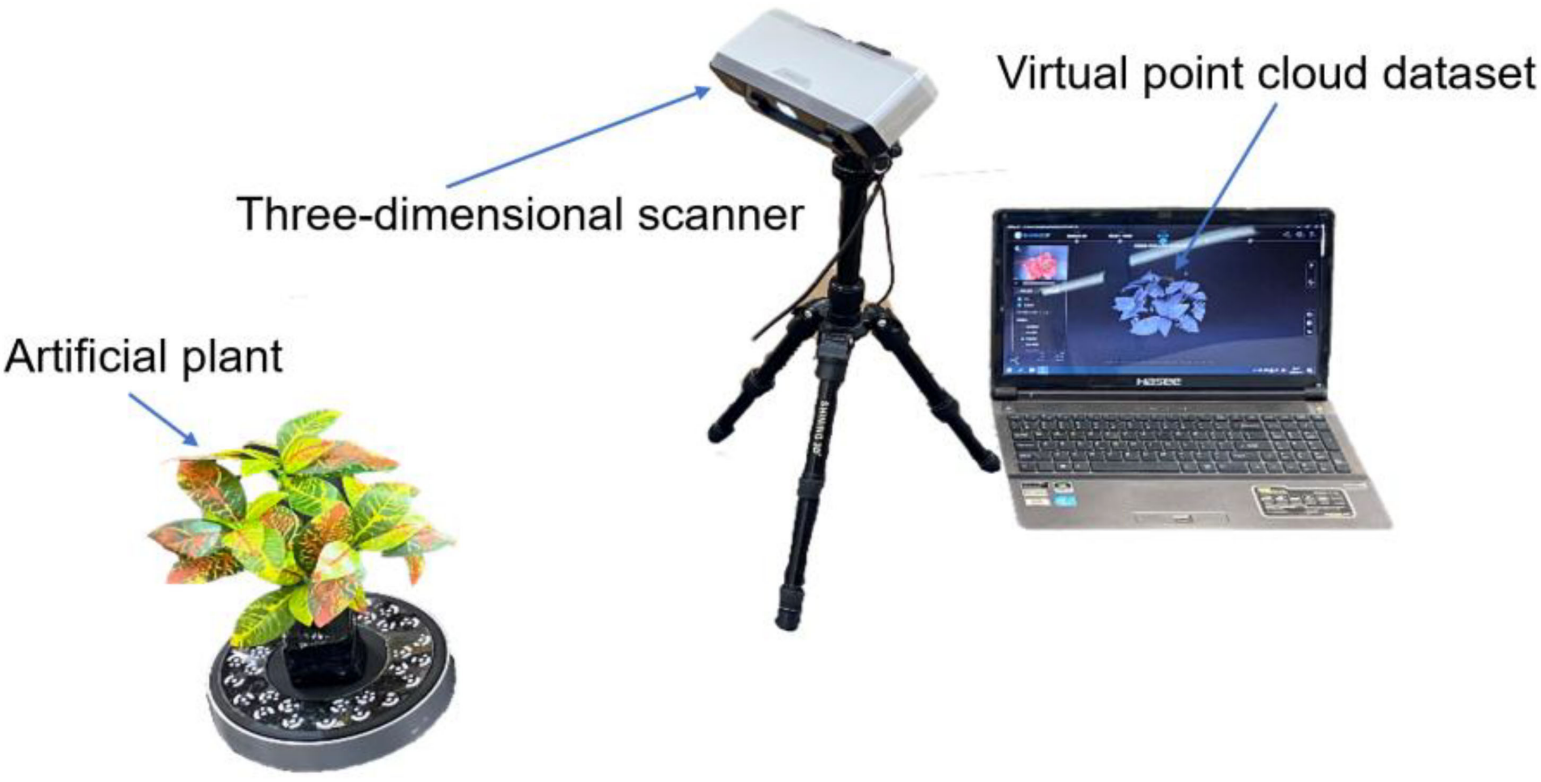
Three-dimensional scanner scanning of artificial plant physical model.

The dataset for this experiment comprised point cloud data samples acquired through the 3D scanning process of a diverse range of objects, including cartons and simulated plants, as illustrated in **Figure 4**. The 3D scanning method effectively captures the point cloud data of these objects in various sizes.

The collected 3D point cloud data undergone a processing step, utilizing (Equation 9) to calculate the offset in each direction (x, y, z). This offset was then subtracted from the entire dataset, resulting in the center of the point cloud data being realigned to the coordinate origin. Consequently, this process ensured the centralization of the complete dataset.

(9) $$\begin{array}{c} \{\begin{array}{c} X_{\text{m}}=X_{\min}+(\frac{X_{\max}-X_{\min}}{2})\\ Y_{\text{m}}=Y_{\min}+(\frac{Y_{\max}-Y_{\min}}{2})\\ Z_{\text{m}}=Z_{\min}+(\frac{Z_{\max}-Z_{\min}}{2}) \end{array} \end{array}$$

In the normalization process, the maximum value (*M*_max_) from the longest axis across the *x*, *y*, and *z* directions of the point cloud data was identified. This maximum value was then used to normalize all the points within the dataset, effectively bounding the data within the (-1, 1) range. To generate a robust dataset, the normalized data was subject to augmentation techniques, involving random rotations about the coordinate center across the *x*, *y*, and *z* axes. This allowed for the acquisition of point cloud models from various perspectives, enhancing the diversity of the dataset. In keeping with practical applications, the study incorporated 3D point cloud data collected. This data served as the incomplete input with a resolution of 2048 points within the network. Post-reconstruction and augmentation, this 3D point cloud data formed the complete dataset used in this study.

#### 2.2.3 Evaluation metrics for shape completion performance

To evaluate shape completion performance for the paired complete and incomplete data, methodologies from ShapeInversion (Lin et al., 2017) were utilized, incorporating CD and *F*_1_ scores as metrics. The CD measures the average minimum distance between the generated point cloud and the ground truth, with larger values suggesting greater discrepancies and smaller values indicating better reconstruction accuracy. The *F*_1_ score, a harmonic mean of precision and recall, provided an overall measure of model performance. For real-world datasets that lack ground truth data and consist only of incomplete inputs, Unidirectional Chamfer Distance (UCD) and Unidirectional Hausdorff Distance (UHD) were adopted as evaluative metrics (Lin et al., 2017). Both metrics provided a measure of similarity between the reconstructed and incomplete point cloud datasets, with smaller distances suggesting better alignment with the original incomplete data.

#### 2.2.4 Implementation and configuration details of the MRC-net network

The MRC-Net network developed in this study operated on a Linux server and is primarily implemented using PyTorch (version 1.2.0) and Python (version 3.7). Experiments were conducted on a server equipped with a Quadro RTX 5000 graphics card. During the pre-training phase, the ShapeNet dataset served as the primary dataset and the ADAM optimizer is employed with an initial learning rate of 0.0001. The batch size was configured to 64 and the number of epochs is set to 2000. This study focused on enhancing precision by training individual models specific to each object class. Within the generative network, the batch size was configured to 1, allowing for the output of completion results sequentially. For all cases, the final complete point cloud resolution output by the generative network was 2048. The configuration of these parameters ensures the reliable and efficient operation of the MRC-Net, yielding high-quality results.

## 3 Results and discussion

### 3.1 Visualization and efficacy evaluation of MRC-net across diverse object categories

**Figure 5:**
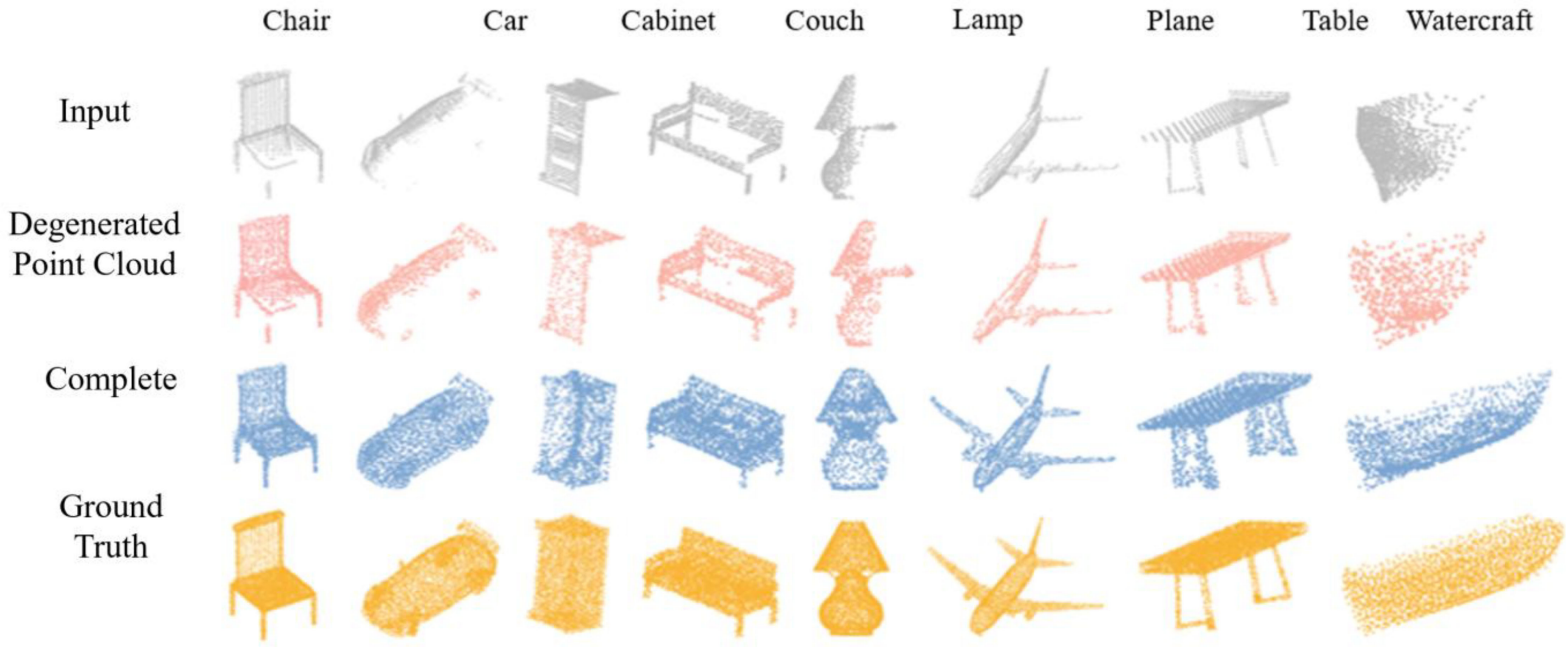
The performance of MRC-Net for eight different object types.

**Figure 5** displays the completion results of the proposed MRC-Net across eight varied categories: chair, car, cabinet, couch, lamp, plane, table, and watercraft. The representation emphasized the proficiency of MRC-Net in accurately reconstructing these diverse objects, capturing intricate details. By employing a consistent dataset for both training and testing phases, the evaluation of MRC-Net’s efficacy was ensured to be impartial and grounded. This dataset, supplied by CRN (Wang et al., 2020), established a level playing field for comparisons with multiple existing supervised and unsupervised techniques under identical virtual scanning conditions. The sequence demonstrated in the figure initiated with the input of fragmented point clouds for the mentioned categories, progresses through an intermediate degradation phase, and culminates in the generation of the finalized complete point clouds. This sequence vividly validated the ability of the proposed method to adeptly complete point clouds.

### 3.2 Comparative performance analysis of MRC-net in point cloud completion on virtual dataset

The performance of the proposed MRC-Net was evaluated using a virtual dataset. For a comprehensive understanding, its efficacy was benchmarked against several established point cloud completion techniques, encompassing both supervised and unsupervised approaches. The central goal of this study was to assess the quality of the completed point clouds, focusing on their detail, overall structure, and other crucial facets. Such evaluations are critical for determining the capability of MRC-Net in addressing missing sections. The CD and F1 scores served as key performance metrics, providing a quantitative measure of the completion outcomes. As presented in **Table 1**, the results of the supervised MRC-Net were described using the format [CD↓/F1↑], with CD values scaled by 10^4^ for clarity. Lower CD values indicated superior reconstruction quality, whereas higher F1 scores signified improved accuracy and completeness of the reconstructed point cloud. MRC-Net’s average CD values were found to be comparable with other supervised techniques and significantly surpassed results from unsupervised pcl2pc methods. Its slight edge over the ShapeInversion technique could be ascribed to its multi-stage refinement mechanism.

**Table 1:** Shape completion results from supervised network. Metrics are reported in the format [CD↓/F1↑] where CD values are multiplied by 10^4^.

Category (object class)	Point cloud completion algorithm			
	PCN (Yuan et al., 2018)	TopNet (Tchapmi et al., 2019)	MSN (Liu et al., 2020)	CRN (Wang et al., 2020)
Chair	11.0/86.0	13.4/82.3	10.6/86.8	8.8/89.7
Car	6.4/94.0	7.8/91.3	7.1/92.3	6.2/93.8
Cabinet	11.3/86.4	12.9/84.1	12.5/85.5	11.4/86.2
couch	11.5/85.2	16.0/80.8	12.0/83.3	11.3/85.1
Lamp	11.6/84.6	14.8/79.4	9.3/88.6	8.5/90.2
Plane	3.5/96.5	4.1/96.0	2.9/97.4	2.3/98.3
Table	10.4/89.4	12.9/85.7	9.6/91.3	9.3/92.9
Watercraft	7.4/91.7	8.9/89.3	6.5/93.1	6.1/94.2
Average	10.1/87.0	11.4/86.1	11.4/86.1	8.0/91.3

From **Table 1**, it was observed that the CRN method consistently outperformed other techniques, denoting its robustness in shape reconstruction across varied objects. Categories such as ‘plane’ were easily reconstructed by most methods, whereas ‘lamp’ and ‘cabinet’ proved challenging, as reflected by their lower F1 scores. Yet, most methodologies achieved an F1 score above 80%, emphasizing their effectiveness in point cloud completion tasks. Collectively, while multiple techniques demonstrated notable proficiency, CRN stood out across numerous categories. It was also evident that the reconstruction’s effectiveness was subject to the inherent intricacies of different objects. **Table 2** detailed the outcomes of unsupervised methods. Performance was gauged using CD, where lower scores were optimal, and the F1 score, with higher values preferred. The Pcl2pcl, as proposed by Chen et al. (2019), generally lagged in precision compared to other methods. In contrast, ShapeInversion, developed by Zhang et al. (2021), showcased notable improvements over Pcl2pcl, especially in the ‘Cabinet’ and ‘Chair’ categories. Yet, MRC-Net outdid both, presenting reduced CD values and superior F1 scores. For categories like ‘Plane’ and ‘Lamp’, MRC-Net’s advancements were particularly evident. On average, its results, with a CD of 13.9 and an F1 of 84.4%, proved its dominance in unsupervised shape completion. The MRC-Net was validated as the preeminent method in this comparative assessment, manifesting the strength and adaptability of its foundational algorithm.

**Table 2:** Effect of unsupervised network shape complementation.

Category (object class)	Point cloud completion algorithm		
	Pcl2pcl (Chen et al., 2019)	ShapeInversion (Zhang et al., 2021)	MRC-Net
air	26.9/70.4	15.4/81.2	**15.3**/**81.4**
Car	15.8/80.0	13.0/85.8	**12.0**/**85.8**
Cabinet	27.1/68.4	16.1/77.2	**15.6**/**78.2**
Couch	34.1/58.4	24.6/78.4	**21.4**/**78.5**
Lamp	25.7/70.4	18.0/81.7	**16.8**/**82.7**
Plane	9.8/89.1	5.6/89.1	**4.1**/**95.7**
Table	23.6/79.0	16.2/85.5	**15.**7/**86.3**
Watercraft	7.4/91.7	10.1/87.0	**9.9**/**86.8**
Average	22.4/74.2	14.9/83.9	**13.9**/**84.4**

Values are reported as [CD↓/F1↑] (CD scaled by 10⁴). Bold indicates the best result per class for each metric.

It could be observed a clear indication of the efficacy of the CRN method in shape reconstruction across various objects. While its consistent performance across categories was desirable, it was essential to recognize the nuances and limitations associated with it, especially when compared to results focusing on unsupervised methods. MRC-Net, showcased exceptional results in unsupervised shape completion. This distinction was crucial given the industry’s increasing emphasis on unsupervised learning methodologies. Despite the superior performance of CRN, MRC was the recommended approach. There exist inherent challenges with supervised learning. The need for paired incomplete and complete datasets for training makes their application in real-world scenarios challenging. CRN, being more reliant on supervised techniques, might be less versatile in unanticipated scenarios due to its dependence on specific training data. However, the versatility unsupervised learning was greater than supervised one. The strength of MRC-Net lies in its foundational algorithm that adapts to unsupervised shape completion. In situations where obtaining paired datasets became challenging, MRC-Net offers a solution that is more flexible and adaptable. Moreover, in terms of generalization capacity, supervised methods often had limited generalization capabilities. This limitation implied that while CRN might excel in specific, known scenarios, it might not be as effective in diverse or unanticipated situations. In contrast, MRC-Net, with its unsupervised learning foundation, offered a broader applicability range, making it more suitable for dynamic real-world applications. In addition, the industry’s trajectory appears to be favoring advancements in unsupervised solutions due to the inherent limitations of real-world scanning data and potential for geometric structure loss. Recommending MRC aligned with this trajectory and emphasizes the importance of continuous research in unsupervised methodologies. Therefore, while CRN demonstrates superior results in specific controlled scenarios, MRC-Net’s adaptability, versatility, and alignment with industry trends make it a recommended choice for point cloud completion tasks.

**Figure 6:**
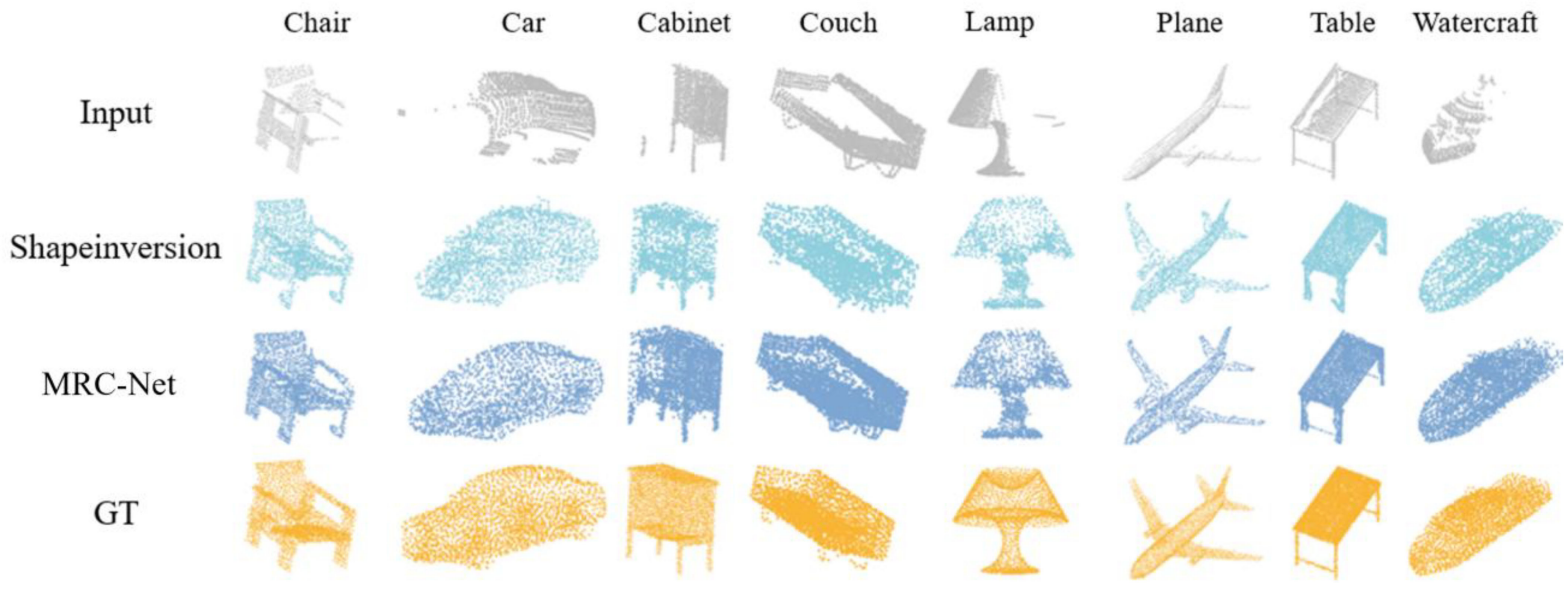
A visualization of the point cloud completion results attained by MRC-Net on the virtual scanning dataset.

In the findings presented, an enhanced method’s performance was rigorously assessed against the established ShapeInversion technique, specifically regarding the completion of structures featuring intricate characteristics. As discernibly illustrated in **Figure 6**, across the eight categories of the CRN dataset, this improved methodology was observed to markedly outperform ShapeInversion. A meticulous scrutiny, particularly into categories such as cabinets, chairs, and cars, brought to light its superior proficiency both in the distribution of points encompassing the overall structure and in the point density within more nuanced regions. Delving further into specific categories, such as couches and tables, revealed even more nuanced insights. When presented with input point clouds of atypically structured couches, the ShapeInversion technique was found to proficiently supplement the upper portions of the couches, yet neglecting the foundational support frames beneath. Conversely, the introduced method not only exhibited a comprehensive completion of the entire couch structure but also displayed a keen detection and reconstruction of the distinctive, brief protruding support frame at the base. This advantageous performance continuity was also identified within the table category, where the proposed approach was observed to equally emphasize the table’s surface as well as the often-overlooked crossbeam. Such meticulous attention ensures a harmonious equilibrium between the generation of intricate details and the creation of the overarching structure. The obtained results validated MRC-Net’s elevated competence in furnishing highly precise and detail-oriented structure completion, especially when confronted with multifaceted and detailed entities, when juxtaposed against alternative techniques.

### 3.3 Analysis of comparative experiments on real datasets

**Figure 7:**
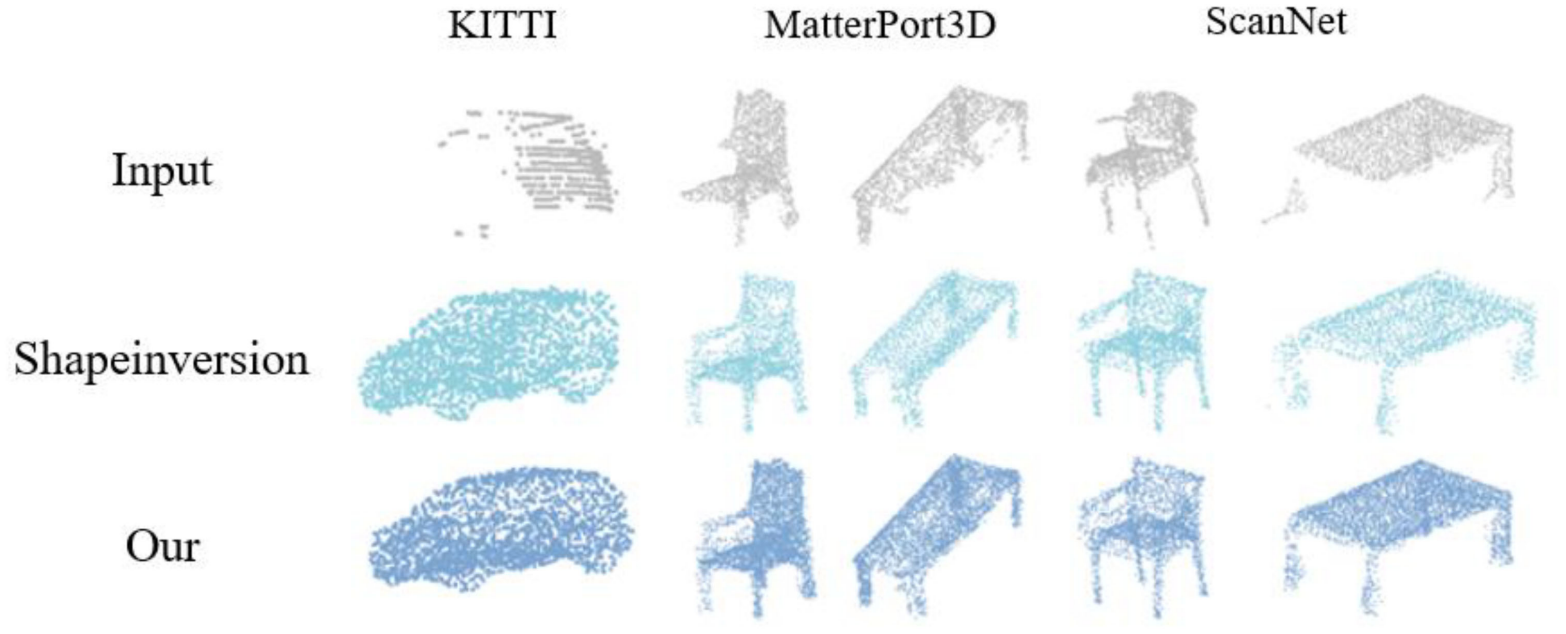
Comparative analysis of point cloud completion in real-world scanned datasets: ShapeInversion *vs*. proposed method.

The quality of data obtained through real-world scanning was often affected by factors such as environment and equipment, resulting in excessive noise and significant missing information. Qualitative comparisons on real-world scans are shown in **Figure 7**, and the corresponding quantitative results—evaluated using UCD and UHD are summarized in **Table 3**. Therefore, this study further employed the method developed within it for real-world scan datasets ScanNet (Lin et al., 2017), MatterPort (Chang et al., 2017), KITTI (Geiger et al., 2012) and compares it with existing methods. It was notable that most of the existing methods primarily focus on generating the overall geometric structure, while the method proposed in this study places greater emphasis on the generation of geometric structural details. The comparison experiment results were quantitatively evaluated using UCD (Unidirectional Chamfer Distance) and UHD (Unidirectional Hausdorff Distance) (Choy et al., 2016; Zhang et al., 2021). The experimental results showed that the method proposed in this study was generally superior to other existing methods, particularly in terms of the reliability of the generated structures. **Figure 7** displays the visual comparison of completion results on real-world scanned datasets. For the chair and table examples in MatterPort, it could be observed that the Shapeinversion method achieves fairly ideal completion results in terms of the overall structure of the chairs and tables. However, there were holes and missing parts of varying degrees, particularly in the chair’s backrest and the corners of the table. The method proposed in this study handled these types of issues quite effectively. In the case of the ScanNet dataset for chairs and tables, the ShapeInversion method appears to lack the connection between the incomplete chair input and the armrests when generating chairs. Additionally, there are excess parts generated at the upper left corner leg of the table. Compared to the method proposed in this study, the reliability and realism of the generated structures were superior to those of ShapeInversion. The method proposed in this study demonstrates better performance in terms of handling detailed structural components and generating more reliable and realistic structures, especially in datasets with real-world scans that often have noisy and incomplete data.

**Table 3:** Complementary results for the real dataset. The results were evaluated using [UCD↓/UHD↓] with UCD scaled by 10^4^,UHD scaled by 10^2^.

Point cloud completion algorithm	ScanNet		MatterPort		KITTI
	Chair	Table	Chair	Table	Car
pcl2pcl (Chen et al., 2019)	17.3/10.1	9.1/11.8	15.9/10.5	6.0/11.8	9.2/14.1
ShapeInversion (Zhang et al., 2021)	4.0/9.3	6.6/11.0	4.5/9.5	5.7/10.7	5.3/12.5
MRC-Net	**3.5**/**10.0**	**3.0**/**12.7**	**4.0**/**8.8**	**4.1**/**11.4**	**4.2**/**8.7**

Values are reported as [UCD↓/UHD↓] (UCD ×10⁴, UHD ×10²). Bold indicates the best (lowest) value per dataset/category.

### 3.4 Performance assessment and practical application of the revised MRC-net algorithm

**Figure 8:**
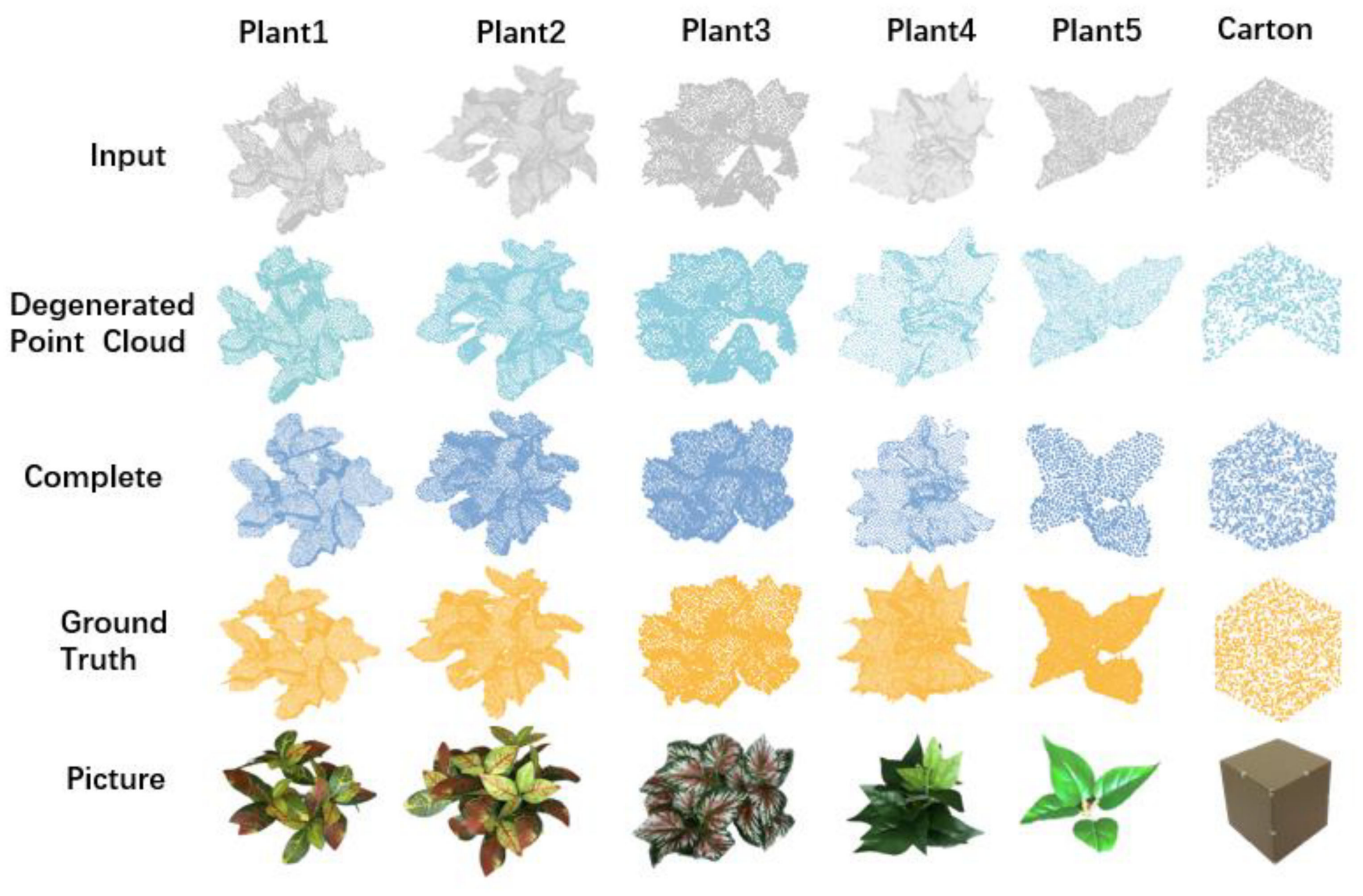
Comparative analysis of MRC-net algorithm performance on standard and irregular objects.

In the experiments undertaken, a revised algorithm was assessed against its predecessor, specifically focusing on detail generation. The results indicated that the enhanced algorithm exhibited superior performance. To validate the algorithm’s practical application, tests were conducted using datasets created from standard cartons and artificially simulated plants. As can be inferred from **Figure 8**; **Table 4**, the updated MRC-Net algorithm, when applied to datasets of standard objects, showed impressive results, evidenced by average CD and F1 values of 3.3 and 97.3, respectively. Upon a detailed analysis of the edge completions for simulated plants, it was noted that the algorithm performed desirable for plants with straightforward external contours. In contrast, for those with more complex exteriors, minor discrepancies in edge completion were detected, yet the integrity of the overall structure was retained. Additional data from **Table 4** revealed the algorithm’s resilience and efficacy, even when applied to plants with intricate contours, demonstrated by average CD and F1 scores of 8.6 and 88.1, respectively. In essence, the findings affirmed the enhanced capability of the point cloud completion deep learning model, introduced in this research, to adeptly complete irregular structures.

**Table 4:** The shape completion effects of cartons and simulated plants on the dataset benchmark.

Point cloud completion algorithm	MRC-Net	
Carton and artificial plant dataset	Carton	3.3/97.3
	Plant1	9.6/87.5
	Plant2	9.0/86.3
	Plant3	9.3/86.8
	Plant4	8.3/88.0
	Plant5	7.2/91.9
	Average	7.8/89.6

The numbers shown in the table are [CD↓/F1↑], where CD is multiplied by 10^4^.

## 4 Conclusions

This research introduced and validated the MRC-Net, an enhanced unsupervised network model predicated on ShapeInversion. Building upon the principles of GAN inversion, MRC-Net innovatively integrates a multi-resolution degradation mechanism with multi-scale discriminators. This design enabled the model to effectively reconstruct both the global structure and intricate details of 3D objects from incomplete point clouds, addressing a significant limitation of prior models which often neglect fine-grained features.

The superiority of MRC-Net was substantiated through comprehensive experiments. On virtual datasets, the proposed network demonstrated performance comparable to existing supervised methods, achieving an average CD of 8.0 and an F1 score of 91.3. Furthermore, its practical efficacy was validated on custom datasets, where it exhibited exceptional proficiency in completing regular objects (cartons: CD 3.3, F1 97.3) and maintained high structural integrity for complex, irregular objects (simulated plants: CD 8.7, F1 88.1). These results confirm that MRC-Net offers a high-performance, stable, and generalizable solution for point cloud completion, providing a reliable data foundation for downstream applications.

Despite strong performance across synthetic and real-scan benchmarks, MRC-Net still exhibits minor artifacts along highly irregular or noisy boundaries. In addition, the multi-resolution degradation, multi-scale discrimination, and multi-stage objectives introduce non-trivial training and inference costs relative to minimalist baselines. A further methodological limitation is that, in our unsupervised GAN-inversion setting, these components are tightly coupled; destructive, module-removal ablations tend to destabilize optimization and conflate training failure with true marginal utility, yielding analyses that are less informative than holistic comparisons.

Future work will therefore proceed along three axes: efficiency—via model distillation, dynamic-resolution routing, and lighter-weight discriminators; robustness—through richer geometric priors, uncertainty-aware completion, and test-time adaptation to severely degraded scans; and principled component analysis—using larger-scale studies under evaluation protocols that avoid objective degeneracy, alongside expansion to more diverse real-world datasets. We expect these directions to reduce compute requirements, improve boundary fidelity, and broaden the practical applicability of MRC-Net.

## Data Availability

The original contributions presented in the study are included in the article/supplementary material. Further inquiries can be directed to the corresponding author.
